# Speed Management Strategy: Designing an IoT-Based Electric Vehicle Speed Control Monitoring System

**DOI:** 10.3390/s21196670

**Published:** 2021-10-07

**Authors:** Gatera Antoine, Chomora Mikeka, Gaurav Bajpai, Kayalvizhi Jayavel

**Affiliations:** 1 African Center of Excellence in Internet of Things (ACEIoT), College of Science and Technology, University of Rwanda, Kigali 3900, Rwanda; 2 Directorate of Science, Technology and Innovation, Ministry of Education, Lilongwe P/Bag 328, Malawi; chomora@gmail.com; 3 Department of Computer and Software Engineering, College of Science and Technology, University of Rwanda, Kigali 3900, Rwanda; gaurav.bajpai.2014@ieee.org; 4 Department of Networking and Communications, School of Computing, College of Engineering and Technology, SRM Institute of Science and Technology, Kattankulathur 603203, India; kayalvij@srmist.edu.in

**Keywords:** electric vehicle, Internet of Things, road safety, speed adaptation, variable speed limit

## Abstract

Road accidents represent the greatest public health burden in the world. Road traffic accidents have been on the rise in Rwanda for several years. Speed has been identified as a core factor in these road accidents. Therefore, understanding road accidents caused by excessive speeding is critical for road safety planning. In this paper, input and out pulse width modulation (PWM) was used to command the metal–oxide–semiconductor field-effect transistor (MOSFET) controller which supplied voltage to the motor. A structural speed control and Internet of Things (IoT)-based online monitoring system was developed to monitor vehicle data in a continuous manner. Two modeling techniques, multiple linear regression (MLR) and random forest (RF) models, were evaluated to find the best model to estimate the required voltage to be supplied to the motors in a particular zone. The built models were evaluated based upon the coefficient of determination $R^{2}$. The RF performs better than the MLR as it reveals a higher $R^{2}$ value and it is found to be 98.8%. Based on the results, the proposed method was proven to significantly reduce the supplied voltage to the motor and consequently increase safety.

## 1. Introduction

Every year, road traffic accidents kill approximately 1.32 million people worldwide, and more than 50 million people sustain non-fatal injuries. Low- and middle-income (LMI) countries account for more than 90% of the world’s fatalities, although they own 60% of the world’s vehicles. The road fatality rate in LMI countries was at 27.5 per 100,000 populations while in high-income countries the road fatality rate was 8.3 per 100,000 [1]. Africa has a fatality rate of 26.6 per 100,000 people while the European region has 9.3 per 100,000 [2]. Road safety brings significant costs to the economy and trauma to society. The average cost of road traffic accidents in LMI countries was estimated to be between 3% and 5% of their gross domestic product (GDP).

**Figure 1:**
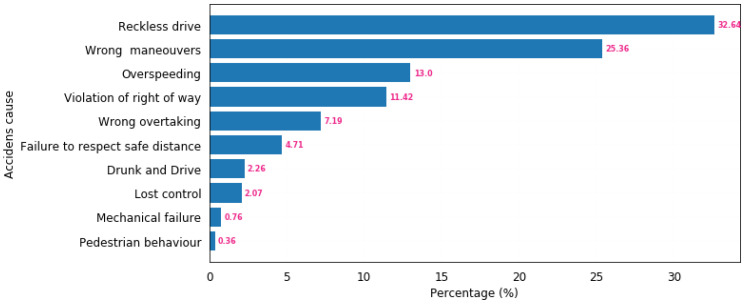
Number of confirmed road accident cases in Rwanda by causing factor (2016–2019).

In Rwanda, motorcycle drivers represent one of the most at-risk occupational groups, with a significant burden of disability-related vehicular incidents and permanent injuries. Among the motorcycle drivers surveyed, 38.7% said they had been in an accident in their lifetime, and 8.5% said they had gone to the hospital [3]. According to the World Health Organization (WHO), global road traffic crashes in 2018 were the 8th and the 1st leading cause of death for children and young adults, respectively. In the same year, the fatality rate per 100,000 people was 29 for Uganda, 34.7 for Burundi, 29.2 for Tanzania, 27.4 for the Democratic Republic of the Congo (DRC), 27.8 for Kenya, and 29.7 for Rwanda. Considering the fatality rate per 100,000 people, Rwanda was ranked 7th in the world [4]. According to the Rwanda National Police (RNP) for the past four years, reckless driving, wrong maneuvering and excessive speeding have been the major causes of traffic accidents. They account for 32.64%, 25.36%, and 13% of traffic accidents, respectively. Figure 1 depicts the cause factors of road accident cases for the past four years. In a typical year, there have been 5595 road accidents on average, with 603 deaths, 789 serious injuries, 1765 minor injuries, and 2437 property damage.

**Figure 2:**
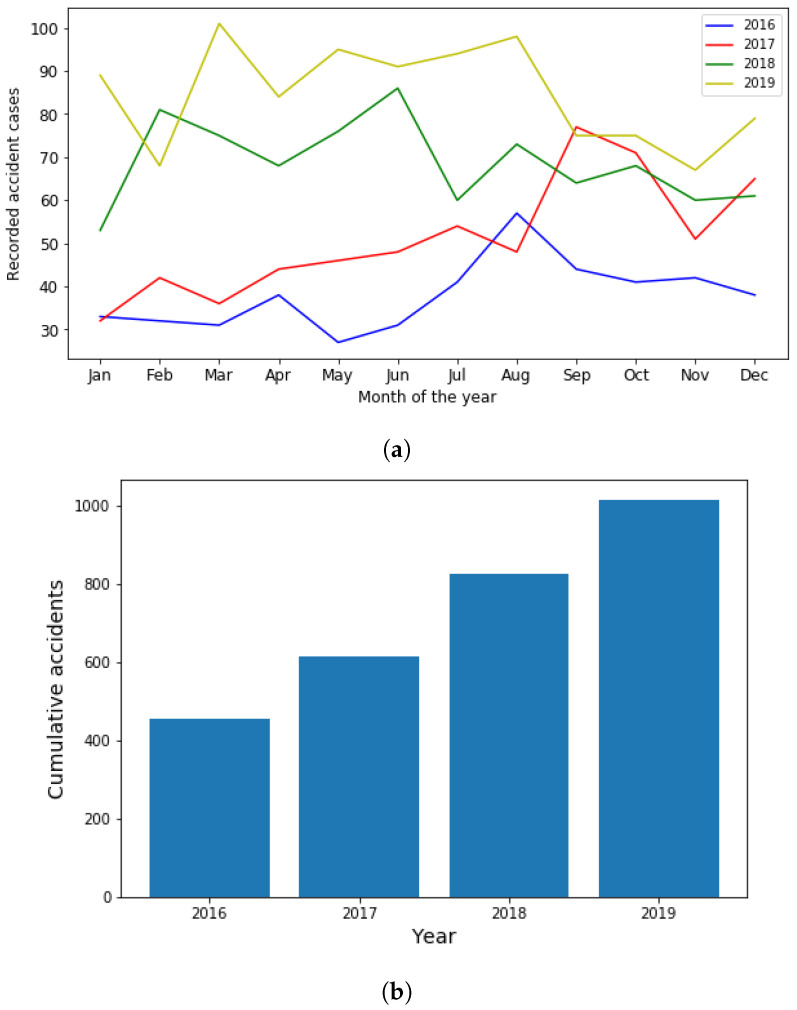
(**a**) Monthly variation of excessive speeding road accidents; and (**b**) cumulative number of excessive speeding due accidents by year (2016–2019).

An increase in average speed is relatively associated with an increase in collision frequency [5]. Figure 2 shows that for the last 4 years, road accidents caused by excessive speeding increased in mean per month with 37.9, 51.1, 68 and 84.6 cases, respectively.

The United States of America (USA) started installing a chip in every entity on the Internet, including vehicles, which will facilitate the Internet of Vehicles (IoV) [6]. The integration of technologies expands the Intelligent Transportation System (ITS) framework, wireless access protocols [7] and GSM [8]. Today’s real-time traffic data available for moving vehicles have enabled the development of ITS applications for traffic control services [9]. The IoT is revolutionizing research by integrating smartness into the existing areas. ITS is tremendously promising to minimize road traffic safety challenges [10]. The IoV allows both collecting and storing vehicles’ movement data in the Cloud [11]. Vehicle data are being sent to the web from any device that can communicate using the representational state transfer application programming interface (REST API) [12]. Building a sensor network for IoT applications implies a higher initial cost.

The GSM cellular network covers 96.4% of Rwanda. The fourth-generation long-term evolution (4G LTE) technology was deployed across 94.2% of its geographic coverage. Developing an application that is cellular communication-based is cost-effective [13]. The in-car navigation system to provide traffic reports through vehicle-to-Cloud communication is crucial in ITS [14]. Police forces along the roads keep continue being employed as the major solution to ensure that road users adhere to the set rules. Nowadays, ITS-based solutions facilitate traveling in a more organized way. One of the approaches has been the variable speed limit (VSL) to optimize traffic flow by adapting the speed limit to real-time conditions [15]. VSL has been used in conjunction with other traffic flow control strategies to improve traffic throughput while reducing bottlenecks [16]. Variable message signs are installed on motorways to notify drivers about the current speed limits [17]. Their contributions are significant in terms of speed harmonization [18]. The presence of an active VSL in the region results in increased traffic density as well as a reduction in flow [19]. The VSL has shown its high influence on traffic flow dynamics such as congestion management [20]. VSLs are being used as an approach to optimize traffic flow in terms of flow harmonization.

The speed of road vehicles is subjected to various parameters such as traffic conditions, vehicle conditions, weather, road curvature, driver behavior, and many others factors [21]. Vehicle speed control can be implemented using the in-vehicle system, e.g., adaptive cruise control (ACC) [22]. Intelligent speed adaptation (ISA) can also be used at a macroscopic level for a whole road segment. Vehicle speed control in related works was based on environment perception [23], road conditions [24], and driver state detection [25]. A gear shift method for the dual clutch transmission (DCT) was proposed for the speed control of automotive motors [26]. However, road traffic accidents are avoidable, and Sub-Saharan African countries lack adequate methods of reporting the tangible and intangible costs of road accidents. This leads to the inappropriate consideration of road accident costs [27]. Human factors related to driving behavior, the driver’s attitude, and driving experience have been demonstrated to have relevant impacts on accident severity [28,29]. Every 1% increase in average speed increases the fatal crash risk by 4%. Hence, there is a need to develop an intelligent system to avoid excessive speeding. Investigating the effect of the change of speed on accidents has shown a strong relationship between speed and road safety [30]. Despite the existing approaches, the variety of factors still challenges the drivers and road accidents keep increasing exponentially. Hence, there is a need to find a reliable and cost-effective system to limit the driver to the specific speed of a particular zone. Speed reduction needs a special focus since it is a priority strategy to reduce the rising fatalities associated with excessive speeding.

This paper was driven by the fact that Rwanda is taking advantage of emerging technologies to address road safety. The transportation sector is one of the major contributors to road safety problems [31]. As a solution, the government has encouraged the use of electric vehicles in its 2050 vision to reduce pollution by up to 38%. Electric vehicles will account for 9% of this target [32]. Various policies are being implemented for the electrification of transport [33]. Electric car sales in 2019 were 2.1 million [34]. Electric vehicles are categorized into battery electric vehicles (BEVs) and hybrid electric vehicles (HEVs) based on the energy type [35]. This paper proposes a dynamic speed adaptation (DAS) method for controlling the operating speed at which the vehicle is observed operating under free flow conditions. Together with the speed sensing module for sensing the maximum lawful speed and predefined road curvature data for a particular zone, the vehicle’s speed limit is adjusted accordingly. The output from the speed limits and road curvature information parameters are presented as explanatory variables to predict the outcome of the response variable, which is the voltage supplied to the electric motor of the vehicle. The data generated by the small setup device was used to evaluate the MLR and RF regression approaches to compute the needed speed limit. The idea of speed control proposed in this paper is based on the working principles of BEVs, where only batteries feed the electric motor. The vehicle thus solely relies on the energy stored in the batteries pack [36].

Motivated by the aforementioned matters, this paper aimed to investigate an adaptive speed control system for road safety. This research provides a data acquisition architecture using IoT technology. It also conducts a comparative analysis of regression models to predict the voltage. The key contributions of this paper are as follows: (i) proposing a hierarchical framework to control the speed and intelligently monitor the vehicle’s data; (ii) validating the efficiency of the proposed speed control system using the developed prototype; (iii) evaluating the effectiveness of the proposed vehicle data logging and monitoring; and (iv) proposing a machine learning model embedded in an in-vehicle device to predict the voltage to be supplied to the motor.

The rest of the paper is organized into the following sections. Section 1 discusses the related literature, Section 2 gives the detailed speed controlling system and an in-depth discussion of the implementation components. Section 3 discusses the experiment and their results. Finally, in Section 4, the conclusion with the future work directions is given.

## 2. Proposed Approach

In this section, a brief overview of the speed sensing and control mechanism is presented. The control parameters and external disturbances that have an influence on the stability of the control system are not considered. The design and implementation of a decision support mechanism with a low level of abstraction are described. The components used for the system validation are presented. Predictive models such as convolutional neural networks, hidden Markov and deep learning techniques were developed to predict the speed based on previous speeding history. In this work, voltage prediction models were built to show the dependence between vehicle speed, voltage supplied to the vehicle’s motor, and road curvature information.

### 2.1. Dynamic Speed Adaptation Architecture

**Figure 3:**
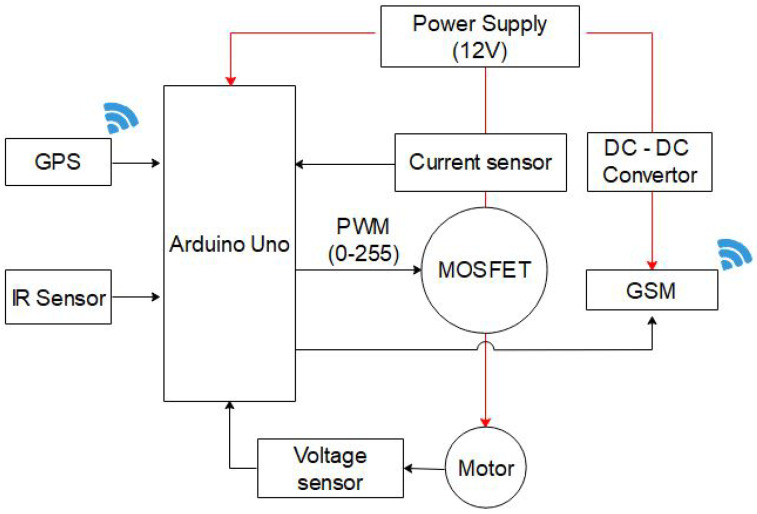
Architecture of the speed control system.

Most drivers perceive posted speed limits as unnatural. This leads them to only reduce speed when the risk of an accident is perceived or when to avoid being caught and punished by road authorities. In order to design, build, and test intelligent VSL control systems, integrated hardware was configured along with the software to be able to operate with different types of sensors and actuators. The sensors range from infrared (IR) sensors, voltage sensors, a Global System for Mobile/General Packet Radio Service (GSM/GPRS) module, MOSFETs, and a DC–DC converter. The microcontroller is programmed to run the controller algorithm, sensor fusion, and serial port communication. The control commands are sent to the MOSFETs that control the voltage supplied to the DC motors. Figure 3 depicts the internal vehicle speed control system components that aid in speed control. Real-time vehicle data are transmitted through GSM/GPRS to the web-server database, assumed to reside in the traffic management center (TMC). The road authorities have the privilege of monitoring vehicle data in a particular zone. Vehicle identification, its geographical location, and speed-related data are transmitted and then stored in the remote database.

**Figure 4:**
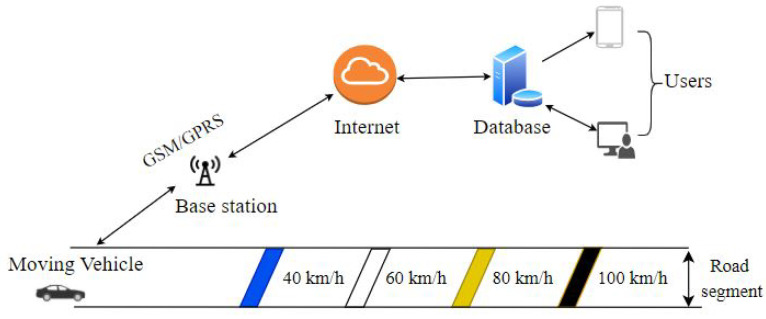
Fundamental layout of IoT-based speed monitoring.

A Cloud-based IoT platform was developed with the primary goal of tracking and storing the data of moving vehicles. System architecture and interfaces are shown in Figure 4.

Developing an IoT-based solution that allows road users and road authorities to track vehicles’ data in real time would contribute to road safety measurements. Setting speed restrictions is one of the strategies for reducing the increase in speed-related accidents. Therefore, it is important to build responsive and effective decision support mechanisms to handle speeding-related issues.

#### 2.1.1. In-Vehicle Setup

**Figure 5:**
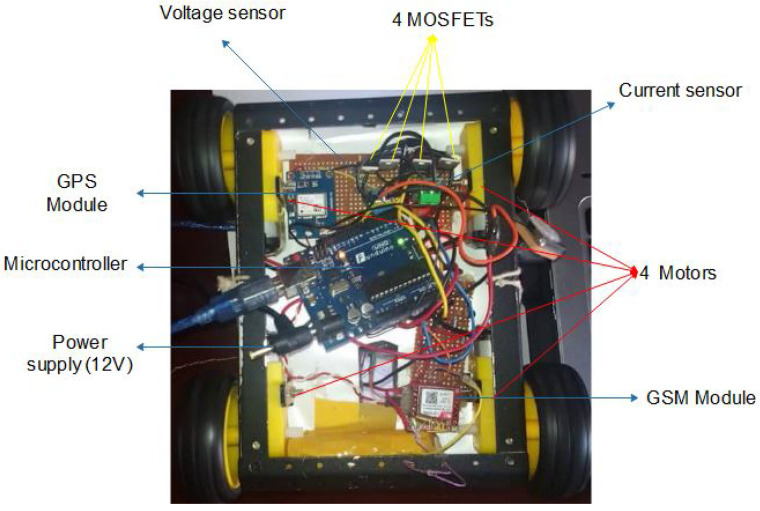
The embedded speed control device.

The in-vehicle device, with the help of the microcontroller, packs the data and then uploads the vehicle’s data to the Cloud web platform through the GSM/GPRS module. The speed of the vehicle, the voltage supplied to the motor, and the current and geolocation data are collected. The microcontroller is programmed to send these data to a remote database. Road authorities and drivers will use various intelligent terminals to access the Cloud platform as well as obtain data in real time. Figure 5 shows the different components required by the moving vehicle.

(1) IR Sensor: infrared sensors are electronic devices that measure infrared radiation in their environment. They have been used to investigate occupancy [37].
(2) Microcontroller: this is the central part of the acquisition of the variable speed control design. The physical programmable board serves as the brain, and the flow chart logic occurs here. The GSM/GPRS module is connected to the microcontroller as it has a set of analogue/digital input/output pins that enable different sensors to be connected to the microcontroller. The GSM/GPRS module transmits the vehicle’s location and details the web storage in real time [38].
(3) GSM/GPRS Module: to allow data transmission to a remote web server, an Arduino Uno module interface is used. This GSM/GPRS module works with GSM frequencies in the range of 850 MHz–1900 MHz. The module, through its protocol, enables data transfer to the database via the GSM network [39].
(4) GPS module: the GPS module compatible with Arduino Uno utilizes data from satellites to locate the moving vehicle at a specific trajectory. The data of the moving vehicle are used to know the exact location of the beginning of the speed limit zone [40]. Data have important applications for road managers and people in transportation research fields such as the detection of the movement of drivers, traffic flows in an area, and predicting the number of accident cases in a specific zone [41].

Color information has been used for the detection of traffic signs [42]. Road sign recognition systems were developed to increase driving safety. However, developing automated methods that recognize the speed limit have been challenged by various factors including dynamics in the environment, uncontrolled illumination caused by solar radiation, camera resolution, and blurred traffic signs [43]. Road sign detection and recognition are the most challenging tasks for the automatic recognition of speed limits. The detection accuracy and recognition rate become challenging when the partial obscuring, blurring, and fading of traffic signs occurs, particularly in a real-time changing environment. Furthermore, fast algorithms during the recognition process and the computational complexity are required [42]. Precise measurements for relative positioning remain challenging when it comes to precise relative tracking results. Global navigation satellite system (GNSS) signals are often blocked in the challenging environments. This leads to the discontinuous carrier phase which has effects on the use of GNSS precise positioning [44].

In order to avoid the failure of the proposed system which might be caused by poor GNNS signal coverage, particularly in multi-layer super ways, a contingency sensor system was used. The IR sensor to measure the reflection of the light for the color painted at the entrance of the speed limit zone was considered. The assumption is that the colors are painted on the road surface. Each color intensity corresponds to a certain speed limit of that particular zone (a voltage that is linearly proportional to light intensity). The IR sensors mounted on the moving vehicle read the road surface and interpret the defined reflected measured wavelengths (the reflection light depends on the color of the surface) when entering the speed limit zone. The information sensed is transferred to the microcontroller for further instructions, interpretation, as well as determining the voltage to be supplied to the motors. The voltage to be supplied to the motors is proportionally assigned to the colors.

**Figure 6:**
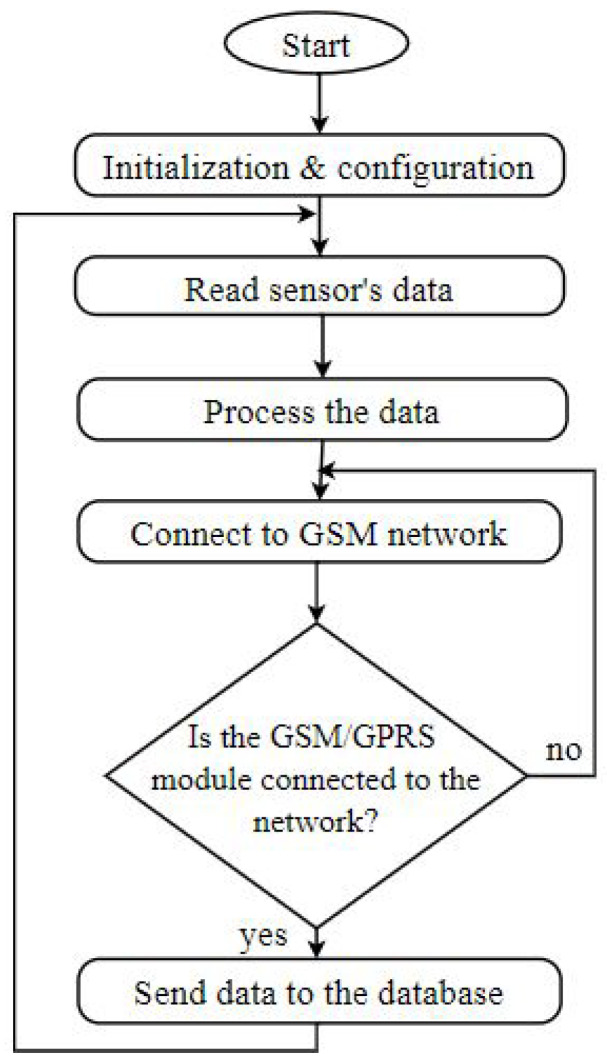
A program running into Arduino Uno.

Figure 6 shows the flowchart of the program that was running on the Arduino Uno microcontroller board. The physical programmable board serves as the brain, and the flow chart logic takes place there.

#### 2.1.2. IoT System Design

**Figure 7:**
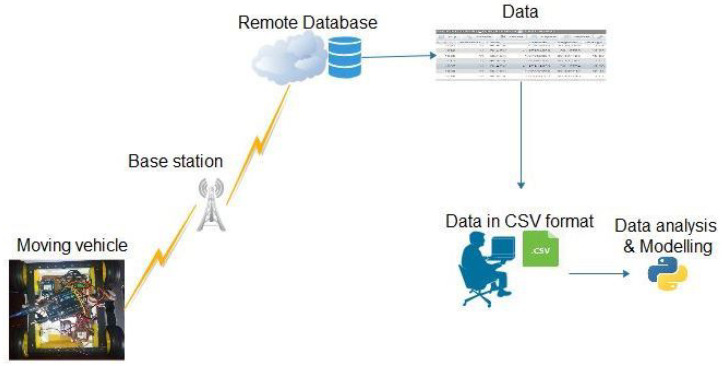
Sample of captured data appealing in the database.

Figure 7 shows the IoT system design. The layers of the architecture are the sensing layer (moving car equipped with the sensors), network connectivity layer (the GSM communication), and service layer (the users). The data collected at the sensing layer are obtained in a variety of formats, including comma-separated values (CSV). Data-based models fully become actionable at this level. Hence, the data might be analyzed to become valuable information to road traffic authorities or health personnel’s specific requirements and patterns. Based on the functional requirements, data-driven models that learn data might be developed to make use of the vehicle’s data [45].

#### 2.1.3. Web Front-End and Web Back-End

The remote database was used to keep the collected vehicle data for data analysis services and user-oriented application services [46]. Different groups of users access all tracking information in real-time from a web-based application. These users may include an admin facility to contact the driver, update color meaning, and the user to track their driving history. This application/service layer is where industry-specific applications such as predictive models can be developed based on a custom application. This module is to be developed using an HTML5 Web app. Cloud servers can be used for planning based on gained knowledge of units (speed versus location, speed versus cases, as well as accident cases versus location) due to faster and flexible data processing features.

### 2.2. Modeling the Supplied Voltage from Battery to the Motor

In driving scenarios, speed is one of the major causes of accidents. Various variables such as road geometry, sight distance, and road surface type were found to have a big contribution to speed. Hence, these factors that influence speed-related accidents were considered to develop DAS systems. Location and geometric information about road curvature [47,48], curve speed warning systems [49], and road curvature with speed limits can be used to adjust the vehicle’s speed [50]. In this work, we proposed a predictive model that can be embedded in the developed smart device installed in the vehicle to keep displaying the speed and inform the internal devices about the voltage to be supplied to the motor. The data used in the modeling are the data collected by the in-vehicle components described in Section 2.1.1. Rwanda follows the policy on the Geometric Design of Highways and Streets [51]. Due to the shortage of data, standards require that curvature is used to set up speed limit [51,52]. The voltage, which is the electrical energy from the car’s batteries supplied to the motors to cause the rotation of the car’s wheels, was predicted. Controlling the operating speed requires following the roadside-imposed maximum speed limit together with the road curvature information. A comparison between the two models, MLR and RF, was performed to find the best model to predict the required voltage to be supplied to the motors. The correlation between voltage with imposed speed limits and road curvature information were transformed to linearize the speeding function. The driver’s operating speed depends on the supplied voltage from the batteries, which in turn depends on the maximum lawful speed posted on the regulatory sign and road curvature information. If the voltage increases, the speed increases. In contrast, the decrease in voltage is equal to the magnitude of the operating speed.

#### 2.2.1. Multiple Linear Regression Model

The MLR technique can be used to model the voltage data for the speed control of the EV in terms of other parameters of the imposed speed limit and the curvature information. For the MLR model, the dependent variable *y* (voltage) is assumed to be a function of *k* independent variables $x_{1},x_{2}\ldots x_{n}$, here referred to as the speed limit and road curvature data. Hence, the model is expressed as

(1) $$y{}_{i}=b{}_{0}+b{}_{1}x{}_{1}+b{}_{2}x{}_{2}+\ldots +b{}_{k}x{}_{i}+e{}_{i},$$

where *y* is an independent variable, $b_{0},b_{1},b_{2}\ldots b_{k}$ are fitting constants, $x_{i}\left(i=1,2,\ldots k\right)$ are predictor variables, and $e_{i}$ is a random error.

#### 2.2.2. Random Forest Model

The RF model is a machine learning model that combines the algorithm of classification to make output predictions from a sequence of regression decision trees. The model is based on the concept of ensemble learning, independently constructed based on a random vector sampled from the input data. Prediction built on the classifier in the assembly. The number of trees in the forest and the number of variables utilized to develop each tree are the two primary characteristics that influence the RF model’s capacity to estimate. The model’s mean square error calculation is calculated by the out of bag (*OOB*) and this is the method for measuring the prediction error. Equation (2) is used to calculate the error:

(2) $${\mathrm{MSE}}_{OOB}=\frac{1}{n}\sum_{i=1}^{n}{\left(O_{i}-P_{iOOB}\right)}^{2},$$

where *n* is the observation number and $P_{iOOB}$ is the average of the *OOB*’s predictions across all the trees.

### 2.3. Validation of the Models

For both the MLR and RF models presented, the informativeness of the models can be considered as sufficient based on the selected number of metrics for evaluating each selected model. In this paper, the training dataset consisted of 70%, whereas the remaining 30% of the records were used for a test. This produces better error results, compared to the 80% testing and 20% training method. To evaluate the performance of both the models, the results were assessed by the mean absolute error (MAE), mean squared error (MSE), root-mean-square error (RMSE), and R squared ($R^{2}$) [53,54,55]. The voltage modeling scenario used in this paper was considered a regression problem, which is a set of statistical processes for estimating the relationships between the response variable and predictor variables, hereby referred to as the posted maximum speed and the road curvature information. The MAE is a risk metric corresponding to the expected value of the absolute error given by the Equation (3). The MSE, which is the average of a set of errors, is given by the Equation (4). The RMSE is the standard deviation of the residuals (prediction errors). RMSE is defined by the Equation (5). The $R^{2}$ is a statistical metric used to measure how much of the outcome to be expected (voltage to be supplied). The $R^{2}$ values range from zero to one [0, 1]. Hence, zero (0) illustrates that the voltage to be supplied to the motor cannot be predicted by the speed and curvature values, while one (1) implies the perfect prediction of both predictors without the error Equation (6):

(3) $$\mathrm{MAE}=\frac{1}{n}\sum_{i=1}^{n}\left|y_{i}-{\hat{y}}_{i}\right|$$

(4) $$\mathrm{MSE}=\frac{1}{n}\sum_{i=1}^{n}{\left|y_{i}-{\hat{y}}_{i}\right|}^{2}$$

(5) $$\mathrm{RMSE}=\sqrt{\frac{\sum_{1}^{n}\left(y_{i}-{\hat{y}}_{i}\right)}{n}}$$

(6) $$R^{2}=1-\frac{\sum_{i=1}^{n}{\left(y_{i}-{\hat{y}}_{i}\right)}^{2}}{\sum_{i=1}^{n}{\left(y_{i}-{\bar{y}}_{i}\right)}^{2}}$$

In Equations (3)–(6), ${\hat{y}}_{i}$ is the predicted value of the *i*th sample and $y_{i}$ is the corresponding true value for the total *n* sample.

## 3. Results and Discussions

### 3.1. Data Acquisition

**Figure 8:**
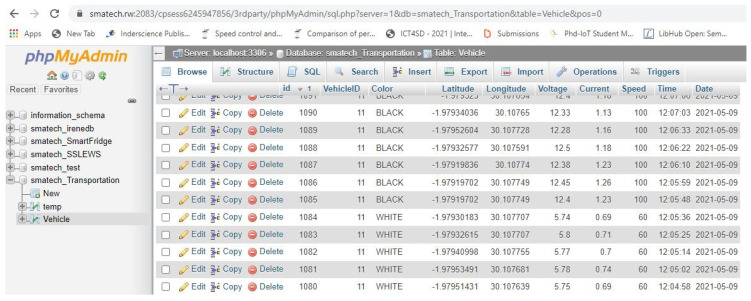
Data presentation in the database.

In the experimental setup configuration, the sensors were connected to a microcontroller board with the GSM/GPRS module enabled to send data to the configured database server. The EV was built with four (4) 12 V batteries linked in parallel. The electric motor only drives the vehicle. The positive and negative terminals of all batteries were connected in the same manner to maintain a constant voltage (12 V). Together with the mentioned sensors, the brain of the prototype is the microcontroller that has the responsibility of regulating the inputs. The MOSFET’s responsibility is to execute the control law. To validate that the designed control system has the desired behavior, a voltage divider rule was used to convert a higher input voltage to a lower output voltage to control the speed of the motor. Input and out pulse width modulation (PWM) generated variable-width pulses to represent the amplitude of an analogue input signal. The flow of the current was controlled by regulating the amount of voltage across the motors, assumed to be measured in kilometers per hour (km/h). The microcontroller uses the PWM technique to control the speed of the motor based on the Equation (7). In order to vary the speed of the motor, the duty cycle of the PWM signal (PWM wave) is on a score of 0-255V. From the Equation (9), the MOSFET changes the amount of applied voltage to the motor that varies in the range of 0–12 V. As detailed in Table 1, a call to analogWrite (100) was applied for speed limit of 40 km/h, analogWrite (150) for 60 km/h, analogWrite (200) for 80 km/h and analogWrite (255) for 100 km/h respectively, such that analogWrite (255) requested a 100% duty cycle equivalent to 12 V. Figure 8 presents the layout of the data transmitted by in the database. Both prediction models were tested using the acquired sensor data captured by the developed IoT system and the curvature data defined in [48].

(7) PWM=(D/256)12V,

where *D* presents the duty cycle, *PW* is the pulse width and *T* is the total period of the signal. However, the duty cycle is calculated by

(8) D=PW/T,

with *T* being the total period of the signal:

(9) vOUT=vIN/(R2/(R1+R2)),

where *vOUT* = output voltage, *vIN* = input voltage and *R*1, *R*2 values of two resistors.

As mentioned previously, the responsibility of the MOSFET controller is to execute the control law by controlling the voltage flow between the batteries and the motor. The simulation scenarios presented in the Table 2, Table 3 and Table 4 were chosen at random with the aim of validating of the functionality of the system.

Furthermore, in these different scenarios, voltage thresholds to obtain the speed limit were required due to some errors that might be caused by the sensors. Furthermore, from experience, the in-vehicle device, when kept running for a long time period, some errors could be produced, consequently affecting the proposed controller performance.

### 3.2. Preparation of Data for Multivariate Forecast Model

Before the ML models, data preparation in Anaconda Python was performed. A separate environment was created by the installation of Keras, Pandas, NumPy, and Matplotlib libraries for data preprocessing and visualization.

### 3.3. Collinearity of the Data

**Figure 9:**
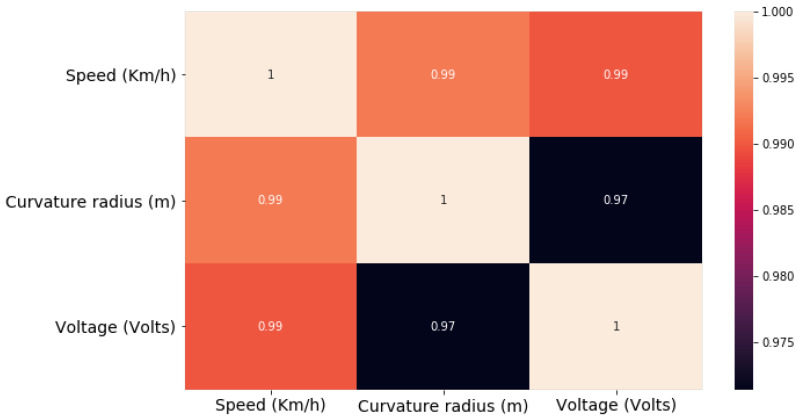
Pearson correlation between speed, voltage, and curvature variables.

The collinearity was computed to assess the correlation between the speed, voltage, and curvature variables. Thus, implying a direct relationship between the discussed variables is necessary to predict the voltage based on the combination of the variables. Figure 9 shows the correlation of the variables. It also shows how all parameters are related to controlling the speed of the vehicle through the quantity of voltage to be supplied to the motors. This reveals that the correlation between speed, curvature of the road, and voltage supplied to the motors occurs in a coincidental manner.

**Figure 10:**
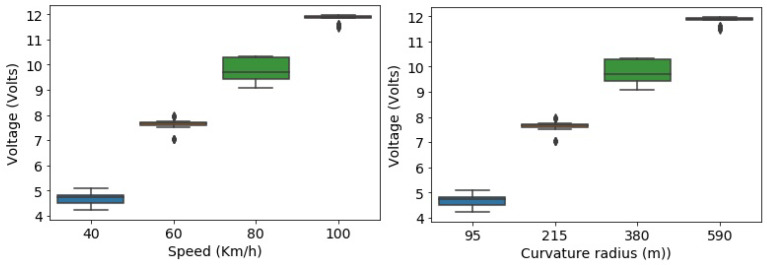
Relationship between variables.

The correlation between both pairs of variables has determined that the correlation between the two variables is high. To control the speed, there is a dependent phenomenon that includes the correlation between voltage and speed. Thus, the results show that the linear regression model to predict the voltage by using the imposed features, i.e., speed limit and curvature, which have a correlation coefficient of *r* = 0.97, and *r* = 0.98, respectively, in the degree of the association of measured variables. Figure 10 indicates that for every positive increase in voltage, the speed increases proportionally. The function box plot in the seaborn library to produce the plots that are used to determine whether the imposed speed limit and road curvature have more voltage outliers.

**Figure 11:**
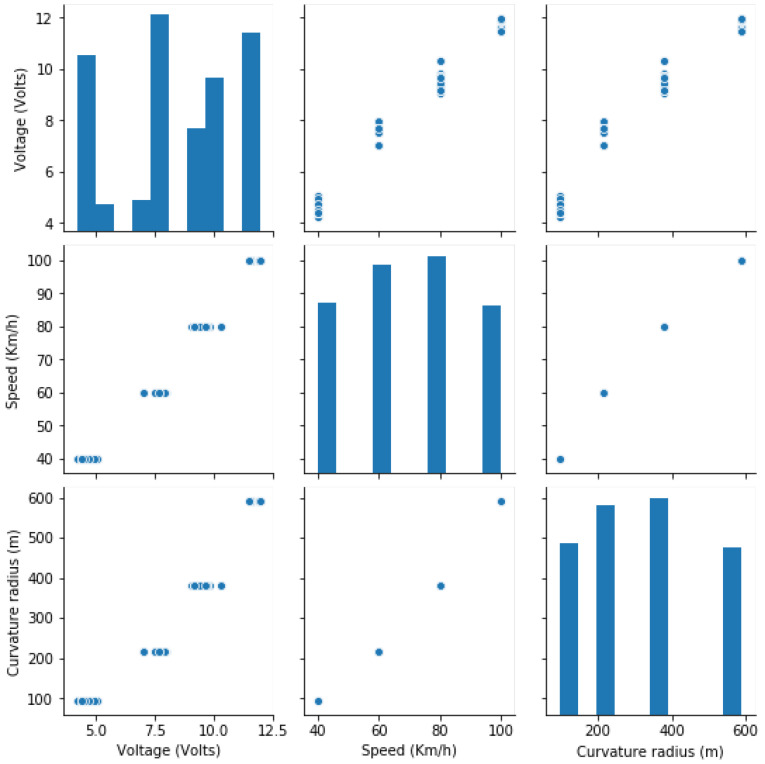
Pair plot of the used variable.

Figure 11 shows the pair’s plot that visualizes the distribution of speed and voltage, as the focus is on reducing speed by reducing the amount of voltage supplied to the motors. Despite the fact that power supply is a significant challenge, the higher the speed, the higher the power supplied to the motor is. This affects the system’s functionality, according to our observations during simulation.

### 3.4. Comparative Analysis of Models

The results of the preferred model were compared for the two machine learning algorithms to evaluate the efficiency of the proposed models. To verify the prediction capability of the proposed models, the evaluation of their results is shown in Table 5. The accuracy of the RF model had a significant improvement over the MLR model. With the highest $R^{2}$ = 0.988 and lowest MAE = 0.194, MSE = 0.066, RMSE = 0.258, respectively, compared to the MLR model which has a lower $R^{2}$ = 0.986 and higher MAE = 0.223, MSE = 0.074, RMSE = 0.273. Hence, the RF-based prediction model showed the highest prediction accuracy in terms of performance with regard to $R^{2}$, with an $R^{2}$ of 98.82% compared to MLR, which has an $R^{2}$ of 98.68%.

Table 6 presents the comparative values for the performance accuracy of the proposed model. The results show that the RF has a high accuracy compared to the MLR model. The table shows the performance values for both the training and test of the proposed models and the optimal parameter values described present the best model.

**Figure 12:**
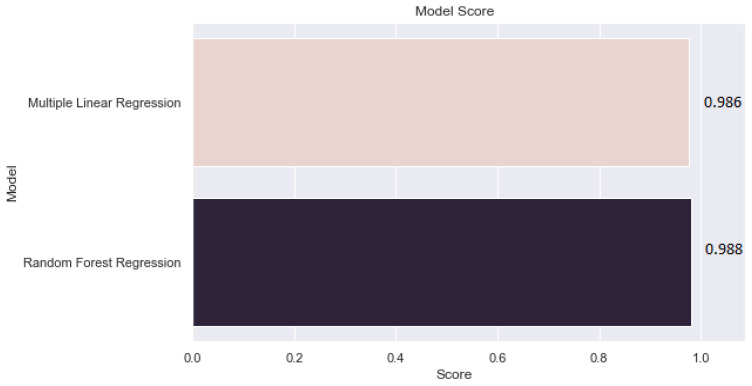
Fitting accuracy: actual and predicted results.

**Figure 13:**
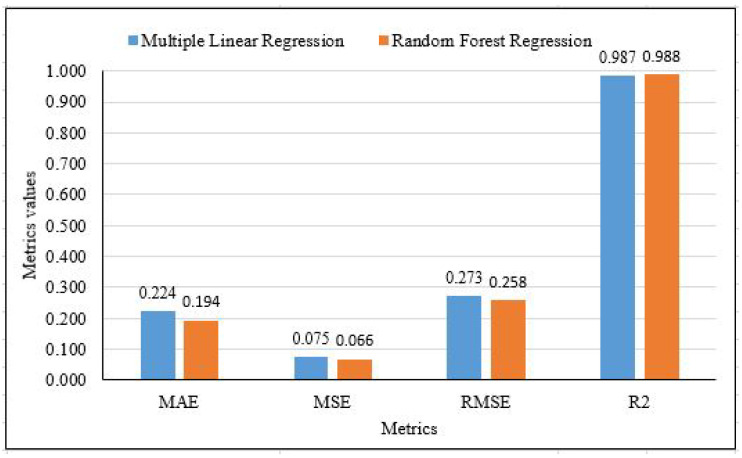
Prediction results: comparison of the RF and MLR model.

Figure 12 and Figure 13 present the result showing that RF model performs well comparing to MLR model.

## 4. Conclusions and Future Works

A data acquisition and communication system based on GSM/GPRS was introduced. The design as well as the execution of all the voltage measures were analyzed in order to maintain speed. Synchronization between software applications and hardware features has been successfully programmed. The transmission synchronization tests carried out demonstrated that the GSM/GPRS-based real-time monitoring was successful. The predictive models of machine learning algorithms, RF and MLR, were applied to the recorded vehicle data to estimate the required voltage per specific speed limit. The RF has proven to be a more powerful modeling approach than the MLR model. The MAE, MSE, RMSE, and higher $R^{2}$ value were more favorable for the RF than for MLR for the prediction of voltage. However, according to our observations during the experiment, there were effects on the system’s functionality when it was kept running for a certain period of time. The voltage sensor would behave unexpectedly. There is an opportunity to extend this work by implementing the method in hybrid vehicles, which combine the use of electric motors and internal combustion engines. The future development will be the integration of several GSM/GPRS terminals with various modeling variables that might interrupt the speed limit. Rain, road damage and unusual events are among these factors. The speed control model proposed demonstrates that the system can be applied to both other systems for parameter monitoring. In this work, the statistical significance of the differences in performance between the three scenarios was not considered, but with potential consideration in future studies.

## Figures and Tables

**Table 1:** Relationship between color, speed, curvature radius and PWM.

Colour	Speed (km/h)	Curvature Radius (m)	PWM
BLUE	40	95	100
WHITE	60	215	150
BROWN	80	380	200
BLACK	100	590	255

**Table 2:** Scenario 1.

Speed	Latitude	Longitude	Voltage	Current	Time	Date
40	−1.9792737	30.107641	4.73	0.4	11:27:47 a.m.	5 May 2021
60	−1.97928962	30.107671	5.74	0.68	11:28:42 a.m.	5 May 2021
80	−1.9793045	30.107688	9.49	0.94	11:29:32 a.m.	5 May 2021
100	−1.97928752	30.107669	12.31	1.4	11:30:13 a.m.	5 May 2021

**Table 3:** Scenario 2.

Speed	Latitude	Longitude	Voltage	Current	Time	Date
40	−1.9793291	30.107719	4.97	0.43	3:21:19 a.m.	8 May 2021
60	−1.9793272	30.107679	5.73	0.71	3:21:08 a.m.	8 May 2021
80	−1.9793045	30.107688	9.48	0.94	3:56:44 a.m.	8 May 2021
100	−1.97946624	30.107625	12.27	1.13	3:20:11 a.m.	8 May 2021

**Table 4:** Scenario 3.

Speed	Latitude	Longitude	Voltage	Current	Time	Date
40	−1.9792737	30.107641	4.89	0.39	11:57:37 a.m.	9 May 2021
60	−1.97951431	30.107639	5.75	0.69	12:04:58 p.m.	9 May 2021
80	−1.9792737	30.107641	9.57	1	12:02:30 p.m.	9 May 2021
100	−1.97932577	30.107591	12.5	1.18	12:06:22 p.m.	9 May 2021

**Table 5:** Fitting accuracy of the data.

**Testing Data**				
**Model**	**MAE**	**MSE**	**RMSE**	$R^{2}$
Multiple Linear Regression	0.223499	0.074573	0.273081	0.986874
Random Forest Regression	0.194704	0.066607	0.258083	0.988276
**Training Data**				
**Model**	**MAE**	**MSE**	**RMSE**	$R^{2}$
Multiple Linear Regression	0.272016	0.114518	0.338406	0.982988
Random Forest Regression	0.254960	0.111780	0.334335	0.983395

**Table 6:** Fitting accuracy of the regression models.

Model	MAE	MSE	RMSE	$R^{2}$
Multiple Linear Regression	0.223500	0.074574	0.273082	0.986874
Random Forest Regression	0.194705	0.066607	0.258084	0.988276

## Data Availability

The data used to support the findings of this study are available from the corresponding author upon request.

## Abbreviations

The following abbreviations are used in this manuscript:

ACEIoT	African Center of Excellence in Internet of Things
ACC	Adaptive Cruise Control
BEV	Battery Electric Vehicle
DAS	Dynamic Speed Adaptation
DC	Direct Current
DCT	Dual Clutch Transmission
GDP	Gross Domestic Product
GSM/GPRS	Global System for Mobile/General Packet Radio Service
HEV	Hybrid Electric Vehicle
ITS	Intelligent Transportation System
IoT	Internet of Things
IoV	Internet of Vehicle
MOSFET	Metal Oxide Semiconductor Field Effect Transistor
RF	Random Forest
RNP	Rwanda National Police
MLR	Multiple Linear Regression
LTE	Long-Term Evolution
LMI	Low-Income and Middle-Income
WHO	World Health Organization
